# Overhearing hushed voices: Using unobtrusive methods to uncover the work-related sentiments of people with epilepsy

**DOI:** 10.1016/j.dialog.2025.100244

**Published:** 2025-10-01

**Authors:** Asha Rao, Surendra Sarnikar

**Affiliations:** Department of Management, California State University East Bay, 25800 Carlos Bee Blvd, Hayward, CA 94542, USA

**Keywords:** Disability, Epilepsy, Unobtrusive research, Stigma, Inclusion

## Abstract

**Purpose:**

Despite high unemployment and discrimination, disability is often overshadowed by race and gender in workplace diversity discussions. This study sought to uncover the work-related sentiments of people with epilepsy (PWE) in their own voices. Epilepsy is a stigmatized invisible disability wherein first-person accounts are difficult to uncover because of stigma. Hence, the discussion on accommodation, inclusion and access for PWE is driven by others, rather than people with epilepsy. By hearing from PWE, workplace solutions can be more relevant to their needs

**Design:**

We use unobtrusive data mining of discussions by PWE by studying anonymous posts on the Epilepsy Foundation community forum over a 16-year period. We examine their sentiments, beliefs, and emotions impacting work for people with epilepsy (PWE), and analyzed perceptions of discrimination to understand what organizations can do better to be inclusive and supportive at work

**Findings:**

We found that PWE sentiments around work were more positive compared to nonwork sentiments. Their work sentiments were analytical, achievement oriented and associated with positive emotions. Work was associated with love and affiliation. However, PWE posts were broadly also more anxious, health centered and tentative reflecting their fears around their health and stigma in employment

**Originality:**

This study is unique in using unobtrusive data mining techniques to analyze the challenges faced by people with disabilities. To our knowledge, there is no such study of adults with epilepsy and the workplace. The methodology used reduces response bias. Questions about disability are difficult to answer for people who face stigma around their disability.

## 1 Introduction

### 1.1 Disability and employment

In the United States, approximately seventy million adults live with a disability [1]. While people with disabilities (PWD) are a vibrant community, they face significant challenges in terms of employment. According to the Centers for Disease Control and Prevention (CDC) [1], one in four Americans, approximately 27 % of the population, has a disability that severely limits their life activities. Only approximately 22.7 % of people with disabilities were employed in 2024, and the employment rate for even those with a college education lagged substantially behind that for people without disabilities [2]. PWD also earn less than others and are paid approximately 12 % less per hour than other employees [3]. Yet, PWD find meaning in work. A meta-analysis of fifty-two studies explored the meaning of work for people with disabilities to find that work was meaningful and important. For PWD, across all types of disability, work was a source of identity, feelings of normality, financial support, and socialization [4].

People with disabilities face many systemic barriers, ranging from inaccessible physical environments and a lack of assistive devices to attitudinal barriers such as prejudice and stigma [5]. Research in this area is complicated in that there are so many varied disabilities, and issues relevant to one group may not be relevant to others. For instance, only 10 % of people with disabilities have mobility impairments and use ramps and the environmental needs of other people with disabilities are not met with ramps. Approximately 26 % of people with disabilities use visible assistive equipment, which leaves the majority (74 %) with relatively invisible illnesses [6]. Invisible disabilities are not immediately apparent to others, which can create hurdles to employment as these individuals may seek to balance any costs of disclosure with their need for accommodation. There is an extraordinarily wide spectrum of invisible disabilities, including Crohn's disease, epilepsy, autism, mental illness, fibromyalgia, chronic fatigue and multiple sclerosis [6].

In this study, we focused on one invisible disability, epilepsy. By doing so, we hope to better operationalize the disability and have greater clarity on its unique challenges by having context specificity to the disability [7]. Currently, epilepsy is one of the most common neurological illnesses in the U.S. Approximately 2.9 million U.S. adults (1 %) have active epilepsy [9]. Historically, people with epilepsy have had remarkable success, with famous epileptics ranging from Alexander the Great, Alfred Nobel, and Teddy Roosevelt to Vincent Van Gogh, Charles Dickens and [the musician] Prince serving as inspirational figures with epilepsy. However, the volume of discrimination cases filed by PWE [8] suggests that very few of these famous characters would thrive in most current organizations.

### 1.2 Epilepsy and work

Epilepsy is a chronic, episodic disorder characterized by a transient neural firestorm in the brain, a surge of electrical activity that causes seizures. Because it is episodic, with fluctuating periods of wellness and illness, PWE face the risk that their invisible disability may become visible beyond their control. There are approximately 3 million Americans with epilepsy [9], and 50 million globally [10]. Reviewing the literature, we find that PWE face both significant unemployment and underemployment [8,11]. Often, ignorance and stigma make disclosure hazardous and debilitating for people with epilepsy, creating a spiral because PWE cannot access the accommodations they need to manage their illness and stay productive.

People with epilepsy (PWE) face enormous challenges in education and work [12]. The unemployment rate for PWE is two to four times greater than that for the general population. They are less likely to have a high school degree than the general population (22.3 % vs 14.6 %). Their adjusted odds of earning a college degree are 30 % lower than those of others. Only 42 % of PWE were employed, compared to 70 % of the population [12].

Despite the prevalence of epilepsy and seizures in the population, research indicates that those diagnosed with this disease experience ignorance, stigma and discrimination in the workplace [13]. The stigma, not just the illness, creates distress, reducing self-efficacy and quality of life. An analysis of Gallup polls over a 25-year period revealed that while prejudice toward PWE declined, 20 % of the population believed that they should not be employed at all [14]. West, Dye and McMohan [8] identified over 5000 cases filed with the EEOC by people with epilepsy regarding discrimination in termination, promotion, disciplinary action and harassment. A survey revealed that 16 % of employers felt they had no jobs for PWE, and a significant 25 % felt that it would be a “major issue” to employ PWE [15]. Overall, people with epilepsy have poorer health, lower levels of personal happiness and life satisfaction and more economic disadvantages than people without epilepsy [13].

Other studies on epilepsy and work point to the meaning of work for people with epilepsy, along with their fears. Clarke, Upton and Castellanos [16] surveyed a sample of 113 respondents with epilepsy, of which approximately 40 % worked full or part-time. They found that unemployed individuals strongly valued work to be “normal” and believed that not having a job was responsible for their lack of independence. Their barriers to work were both their fears of seizures, injury and poor performance as a consequence, and similar fears held by their family. The authors point to an internalization of social stigma and societal fears through adolescence as reducing self-efficacy and the ability to work despite the perceived high status of working. Stigma, seizure severity, and psychosocial variables such as low self-esteem, passive coping style, and low self-efficacy predict employment [17].

The purpose of this study is to identify the sentiments around work for people with epilepsy (PWE) from their **own** perspective and to understand accommodations that firms can provide to allow them to thrive and perform meaningful work.

### 1.3 Unobtrusive methods and sentiment analysis

Given that the existing research indicates that disclosure is low for PWE and that advocacy groups such as Epilepsy Foundation Affiliates advocate limited disclosure, this study seeks to look behind the curtain of concealment and examine work concerns from the perspective of people with epilepsy. In this study, we take a unique approach and use unobtrusive data mining of public discussions by PWE to hear their voices and stories because their anonymity allows for disclosure without stigma, giving them the freedom to speak without fear. Hearing their voices is critical because the perspectives of others, such as employers, rather than PWE, have predominantly shaped the bleak narrative on their employment issues [18]. Anonymous chat or forum data is ecologically valid because participants write in their own words, in their own context, without prompts from researchers.

Anonymity in a public forum dedicated to those with epilepsy helps strip away the layers of concealment for an honest examination of workplace concerns, giving participants a safe space for discussions [19,[29], [30]]. Stone and Colella [7] espouse using unobtrusive methods to study disability to increase accuracy and reduce social desirability bias by managers asked to rate disabled employees. Suler [29] refers to this as the online disinhibition effect where people are more open and honest with anonymity. Joinson [30] found that computer mediated communication encouraged self-disclosure more than face-to-face dialog. We argue that unobtrusive methods are important for increasing the accuracy of responses from PWE themselves by reducing social undesirability bias and fear of stigma.

We take a text analytics-based approach to examine the posts of PWE to identify (a) sentiments around work from their perspective and (b) identify causes of discrimination from their posts. Through a first exploratory pass we look at some of the elements of the broader conceptual framework discussed above that maps a long-term research agenda on epilepsy and work.

In recent years, researchers have used text mining to analyze easily accessible data. For example, Suzuki et al. [20] perform text analysis to analyze attitudes toward mask wearing and Dsouza et al. [21] perform sentiment analysis and content analysis on tweets to generate insights on communication and patterns of stigma related to monkeypox among the LGBTQ+ community. We use sentiment analysis to examine posts on the Epilepsy Foundation discussion forum to identify their sentiments, i.e. the work beliefs, feelings and concerns of importance to PWE, and to examine their perceptions of discrimination. Sentiment analysis technique is a combination of linguistics and computer science that can extract the sentiments underlying thoughts, emotions and opinions from vast sources of data.

## 2 Research methods

### 2.1 Study design and data

Our study is based on anonymous archival data. Since the study did not collect or analyze any personally identifiable information, and did not involve interactions or intervention with individuals, it is not considered human subjects research. Nevertheless, we still reviewed our protocols with our Institutional Review Board and received exempt status for the research study. We used an observational study design with retrospective analysis. Specifically, we analyzed the publicly available user-generated content from the online discussion forum without any intervention or manipulation. Using text mining and sentiment analysis, we quantitatively examined patterns in language use and emotional tone across posts. This approach aligns with observational research principles, where naturalistic data is analyzed to understand patterns and insights without experimental control.

The data for the study was collected from a public community discussion forum on epilepsy available on the Epilepsy Foundation website.^1^ The free and open community discussion forum is a platform for people with epilepsy (PWE) to discuss issues around epilepsy and includes several categories, including medication, therapy, and gender- and age-specific issues. Users self-select the categories and post their messages under one of the categories. Since our primary goal is to study work related concerns, we collected posts under the “Adults Living with Epilepsy” category, which included posts by adults and their caregivers on topics related to managing and living with epilepsy. We excluded forums that were focused on teens and children, or forums focused on medication, care givers, therapies and products. In summary, our inclusion criteria were to include posts on the epilepsy forum that were posted under the category label “Adults Living with Epilepsy” and exclude posts that were posted under any other category. We collected 7142 discussions posted over a span of 16 years from 2004 through 2020. The Epilepsy forum was started in 2004 and retired in 2020. Each post includes a title, main content of the post and the date of the post. On average, each post measured 220 words in length and received an average of 4.5 comments.

We used Linguistic Inquiry and Word Count (LIWC), a computerized text analysis tool that uses a dictionary-based approach for analyzing linguistic and psychological constructs expressed in text data [22]. Topic modeling and computerized text analysis are being increasingly used in management research and have the potential to advance management theory by helping detect novel and emergent phenomena based on an analysis of text data [23]. We chose the LIWC tool for our content analysis since its validity and reliability have been extensively studied in multiple domains [22,24]. Recent examples of LIWC-based computerized text analysis include an analysis of governmental briefings on COVID-19 [25] a study of autism advocacy groups [19] and an analysis of social media conversations about suicide among teens and adults with epilepsy [26]. Detailed reliability and validity statistics for LIWC, including cronbach estimates are provided in [22].

### 2.2 Linguistic analysis

For each forum post, we obtained scores for LIWC variables that represented various linguistic and psychological dimensions. We focus on three summary language descriptors: analytical thinking, authenticity and emotional tone. We assess seven LIWC psychological dimensions including affective processes (positive and negative emotions, anxiety, anger and sadness and two cognitive processes (of tentativeness and certainty); five motivational drives of achievement, power, affiliation, risk and reward. We also include the LIWC dimensions regarding physical wellbeing (health, body) and perception (space and motion), since we are analyzing posts around epilepsy. Finally, we looked into work which LIWC identifies as one of 5 lifestyle dimensions of life.

Each dimension is measured based on a dictionary of terms corresponding to the dimension. For example, anxiety is measured based on the occurrences of associated words such as worried, fearful etc. The scores for each dimension are calculated as the percentage of total words in the sample text that represent the dimension. In Table 1, we include a correlation matrix between the different LIWC variables used in this study. The correlations help us confirm the categories of interest for analysis.

**Table 1:** Correlation Matrix of the LIWC Variables.

	Analytic	Tone	affect	posemo	negemo	anx	anger	sad	tentat	certain	bio	body	health	drives	affiliation	achieve	power	reward	risk	motion	space	work
Analytic	1.00																					
Tone	0.15	1.00																				
affect	−0.04	0.12	1.00																			
posemo	0.08	0.70	0.69	1.00																		
negemo	−0.14	−0.59	0.64	−0.10	1.00																	
anx	−0.12	−0.35	0.34	−0.07	0.54	1.00																
anger	−0.05	−0.26	0.29	−0.05	0.46	0.11	1.00															
sad	−0.03	−0.29	0.36	−0.02	0.53	0.08	0.09	1.00														
tentat	−0.16	0.07	−0.01	0.04	−0.06	−0.03	−0.05	−0.05	1.00													
certain	−0.15	0.03	0.10	0.09	0.05	0.02	0.03	0.01	−0.01	1.00												
bio	0.13	−0.12	−0.10	−0.16	0.04	0.01	−0.01	0.01	−0.03	−0.06	1.00											
body	0.02	−0.17	−0.06	−0.14	0.08	0.03	0.04	0.00	0.00	−0.03	0.55	1.00										
health	0.14	−0.06	−0.09	−0.12	0.00	−0.01	−0.03	0.01	−0.05	−0.06	0.79	0.02	1.00									
drives	0.09	0.12	0.32	0.27	0.15	0.00	0.08	0.12	−0.10	0.00	−0.18	−0.13	−0.14	1.00								
affiliation	0.07	0.27	0.17	0.29	−0.07	−0.06	0.00	−0.01	−0.03	0.02	−0.18	−0.14	−0.14	0.57	1.00							
achieve	0.08	0.09	0.16	0.16	0.05	−0.04	0.01	0.20	−0.04	−0.02	−0.13	−0.11	−0.08	0.48	0.07	1.00						
power	0.11	0.00	0.10	0.05	0.08	0.02	0.11	0.06	−0.09	−0.01	−0.01	0.01	0.01	0.53	0.11	0.09	1.00					
reward	0.01	0.14	0.20	0.23	0.02	−0.01	0.01	0.02	−0.04	0.01	−0.09	−0.06	−0.07	0.48	0.01	0.18	0.05	1.00				
risk	−0.04	−0.25	0.28	−0.02	0.41	0.10	0.04	0.17	−0.02	0.01	−0.01	−0.02	−0.03	0.34	−0.02	0.11	0.00	0.05	1.00			
motion	0.04	−0.07	−0.04	−0.07	0.02	0.02	0.03	0.00	−0.09	0.00	−0.05	0.05	−0.09	0.02	−0.05	0.03	0.05	0.01	−0.01	1.00		
space	0.24	−0.05	−0.07	−0.07	−0.02	−0.05	0.00	0.02	−0.07	−0.06	−0.06	0.12	−0.14	0.07	−0.03	0.02	0.17	0.01	−0.07	0.21	1.00	
time	−0.09	−0.19	−0.13	−0.20	0.04	0.06	0.00	0.03	−0.11	0.10	0.08	0.08	0.05	−0.08	−0.18	−0.02	−0.03	0.00	0.00	0.11	0.08	
work	0.19	0.11	−0.03	0.05	−0.10	−0.07	−0.04	−0.03	−0.09	−0.06	−0.16	−0.14	−0.08	0.23	0.04	0.28	0.20	0.05	0.01	−0.01	−0.03	1.00

Personal concerns related to work is our main variable of interest in this paper. Affective processes capture both positive emotions and negative emotions such as sadness, anger and anxiety expressed within posts. To identify motivating factors related to work, we measured the LWIC drive-related variables, including achievement, affiliation, power, risk and reward. Given the physical manifestation of epilepsy in seizures and environmental triggers, we also included categories of space and motion that could be associated with desired accommodations. In addition to the above categories, we also measure the overall language variables that estimate the analytical thinking and emotional tone reflected in the posts.

### 2.3 Statistical analysis

We conducted multiple levels of analysis that we will outline and then elaborate for clarity. First, we made a comparison of posts on the epilepsy forum with benchmark corpora to understand how this sample differs from the standard. Second, a comparison between work and nonwork posts by PWE on the forum was performed to understand how work sentiments differed from general sentiments of PWE. Third, we isolated a subset of posts that mentioned discrimination and manually coded them so as to focus on their perceptions of discrimination.

We compared the mean values of the selected variables in Epilepsy Forum posts with three different benchmark corpora, to identify the unique characteristics of the Epilepsy Forum posts and understand how they differ from generic social media posts in terms of the various psychological constructs expressed in the posts. Specifically, we analyzed the LIWC variable scores for Twitter corpora, a corpus of blog posts, and an expressive writing corpus consisting of essays on deeply emotional topics written by a variety of individuals provided with the LIWC program [22]. The Twitter, blog post, and an expressive writing corpus benchmark corpora provide multiple contexts with general posts of varying length from very short (twitter) to larger posts (blogs) and essays on emotional topics (expressive writing corpus). This allows us to compare the forum posts by PWE to the benchmark corpora and identify variations in emotion and sentiments expressed by PWE. The benchmark corpora have been provided with the LIWC program allowing for comparison across different studies.

We analyzed the distribution of the variables using visual analysis and the Anderson-Darling tests to test for normality of the data. However, since the data were not normally distributed, we used the Wilcoxon test, a non-parametric alternative to one sample *t*-test, to identify cases where the category means were significantly different from those of the baseline corpora. We also applied the Bonferroni adjustment to correct *p* values to account for multiple comparisons. We then split the Epilepsy Forum posts into work-related and nonwork-related posts to identify key differences between work-related and nonwork-related posts. We used the LIWC scores for the work category to identify work and non-work-related posts. Specifically, all posts that had a work score of one standard deviation above mean work score were identified as work-related posts and others were labeled as non-work-related posts. The difference in category means between work and nonwork-related posts was tested using a two-sample Wilcoxon test with adjustments to *p* values to account for multiple comparisons.

We found very few posts that directly mentioned discrimination. To identify posts related to discrimination, we first searched for all posts that included the word stem “discriminat” to capture variations such as discriminate, discrimination etc. We coded these along the 4 categories identified by West et al. [8] in their analysis of allegations of workplace discrimination by PWE. The four categories are getting a job (hiring, advertisement, etc.), working conditions (wages, work hours, disciplinary action, harassment, etc.), keeping the job (termination, reduction in work, etc.), and unclassified (careers. Work related broad questions etc.) The two co-authors first discussed and agreed on a common definition of each of the 4 categories for discrimination related posts. We then independently coded the posts along the 4 categories.

## 3 Results

The results of benchmarking epilepsy forum posts with Twitter, expressive writing and Blog posts are presented in Table 2A and B.

**Table 2:** Comparison of EF characteristics with those of general text corpora.

A					
Category	Blog Means (p values for μ_ep_ – μ_b_ < 0)	Expressive Writing Means (p values for μ_ep_ – μ_ex_ < 0)	Twitter Means (p values for μ_ep_ – μ_t_ < 0)	EP Forum Means	One Sample Wilcox Test at 0.01 level
Analytical	49.89 (p < 2.2e-16)	44.88 (*p* < 2.2e-16)	61.94 (*p* < 2.2e-16)	40.18	Significantly lower than all three benchmarks
Achievement	1.27 (p < 2.2e-16)	1.37 (*p* < 2.2e-16)	1.45 (*p* < 2.2e-16)	1.089	Significantly lower than all three benchmarks
Emotional Tone	54.5 (*p* < 2.2e-16)	38.6 (*p* < 2.2e-16)	72.24 (*p* < 2.2e-16)	32.71	Significantly lower than all three benchmarks
Affective Processes	5.79 (p < 2.2e-16)	4.77 (*p* < 2.2e-16)	7.67 (p < 2.2e-16)	4.34	Significantly lower than all three benchmarks
Positive Emotions	3.66 (p < 2.2e-16)	2.57 (p < 2.2e-16)	5.48 (p < 2.2e-16)	2.14	Significantly lower than all three benchmarks
Negative Emotions	2.06 (p ≈ 1)	2.12 (*p* ≈ 1)	2.14 (p ≈ 1)	2.131	Not significant
Anger	0.68 (p < 2.2e-16)	0.49 (*p* < 2.2e-16)	0.75 (*p* < 2.2e-16)	0.289	Significantly lower than all three benchmarks
Power	2.07 (*p* < 2.2e-16)	2.02 (p < 2.2e-16)	2.17 (p < 2.2e-16)	1.82	Significantly lower than all three benchmarks
Reward	1.49 (*p* < 2.2e-16)	1.56 (*p* < 2.2e-16)	1.86 (p < 2.2e-16)	1.253	Significantly lower than all three benchmarks
Motion	2.15 (p < 2.2e-16)	2.58 (*p* < 2.2e-16)	1.94 (*p* < 2.2e-16)	1.776	Significantly lower than all three benchmarks
Space	6.43 (p < 2.2e-16)	6.96 (p < 2.2e-16)	6.51 (p < 2.2e-16)	5.522	Significantly lower than all three benchmarks
Certainty	1.56 (p < 2.2e-16)	1.51 (p < 2.2e-16)	1.43 (p < 2.2e-16)	1.4	Significantly lower than all three benchmarks
Drives	6.87 (p < 2.2e-16)	7.35 (*p* < 2.2e-16)	7.5 (p < 2.2e-16)	5.59	Significantly lower than all three benchmarks
Affiliation	2.2 (p < 2.2e-16)	2.45 (*p* < 2.2e-16)	2.53 (p < 2.2e-16)	1.283	Significantly lower than all three benchmarks
Work	2.04 (p < 2.2e-16)	2.64 (*p* < 2.2e-16)	2.16 (p < 2.2e-16)	1.59	Significantly lower than all three benchmarks


B					
Category	Blog Means (p values for μ_ep_ – μ_b_ > 0)	Expressive Writing Means (*p* values for μ_ep_ – μ_ex_ > 0)	Twitter Means (p values for μ_ep_ – μ_t_ > 0)	EP Forum Means	One Sample Wilcox Test at 0.01 level
Authentic	60.93 (p < 2.2e-16)	76.01 (*p* < 2.2e-16)	50.39 (*p* < 2.2e-16)	74.99	Significantly higher than Blog and Twitter corpora
Anxiety	0.27 (p < 2.2e-16)	0.5 (p ≈ 1)	0.24 (p < 2.2e-16)	0.576	Significantly higher than Blog and Twitter corpora
Sadness	0.44 (p ≈ 1)	0.5 (*p* ≈ 1)	0.43 (p ≈ 1)	0.454	Not significant
Biological	2.16 (p < 2.2e-16)	2.59 (p < 2.2e-16)	2.6 (p < 2.2e-16)	4.788	Significantly higher than all three benchmarks
Risk	0.46 (p = 3.15e-14)	0.54 (*p* = 0.8813)	0.46 (*p* = 3.15e-14)	0.667	Significantly higher than Blog and Twitter corpora
Body	0.74 (*p* < 1.35e-8)	0.69 (p < 2.2e-16)	0.77 (*p* < 8.5e-4)	1.11	Significantly higher than all three benchmarks
Health	0.61 (p < 2.2e-16)	0.93 (p < 2.2e-16)	0.54 (p < 2.2e-16)	3.386	Significantly higher than all three benchmarks
Tentative	2.82 (p < 2.2e-16)	2.89 (p < 2.2e-16)	2.35 (p < 2.2e-16)	4.382	Significantly higher than all three benchmarks

The results indicate that in comparison to benchmark corpora of expressive writing, blog and Twitter posts, PWE posts on the Epilepsy Forum are significantly less analytical, meaning they are less formal and hierarchical and more informal, personal and narrative according to the LIWC interpretation guidelines [22]. They are also less emotional in tone, meaning the posts have fewer positive emotions overall and greater negative emotions such as sadness. Their posts demonstrate significantly more anxiety and risk taking than expressive writing, blog and Twitter posts. Higher anxiety is referenced with the more frequent use of words such as fear, worry, and expressing tension. Risk taking includes references to dangers, concerns, things to avoid. The score reflects the percentage of total words within the text that have these words. They express significantly lower positive emotions and are not different in their expression of negative emotions of sadness.

PWE posts on body and health were much higher than in benchmark corpora, inducing a strong interest in discussion on how to manage the illness and its symptoms. These include references to the body (e.g. ache, heart) and to health (e.g. medications, seizure symptoms and events).

In examining drive or motivation, PWE posts are significantly lower on motives of achievement, affiliation and reward. Affiliation is captured by references to others; achievement by references to success or failure, striving; reward by references to rewards, goals, incentives. The drive for power includes references to status, dominance and social hierarchies, and risk to dangers, concerns and things to avoid. Unsurprisingly, PWE posts were significantly higher on risk.

Surprisingly, categories of motion and space were significantly lower. Motion references any words around movement such as walking, leaving, going; space refers to spatial orientation such as around, over, above. With the physical impact of a seizure, we assumed these would be salient to PWE. Their posts expressed more cognitive tentativeness which is references in words like “maybe”, “perhaps”, “I guess” and less certainty references in works like “always” and “never”. These can be seen to reflect less self-confidence.

Finally, work is less salient than in other benchmark corpora suggesting that the forum is a place to discuss more personal matters that are likely difficult to share in the formal workplace. Yet the range of personal topics discussed was wide and were relevant to the workplace. For instance, many posts in the biological and health dimensions focused on medicine and the side effects that impact people during work hours, on the importance of driving, the efficacy of cannabis for controlling seizures (which is illegal in many workplaces), being alone during a seizure, being in the presence of others which leads to disclosure.

The category means and the results of the significance test of differences in work/nonwork posts are presented in Table 3.

**Table 3:** Comparison of work- and nonwork-related posts on Efs.

Category	Not Work Related	Work Related	Direction (Work vs Non Work)	Two Sample Wilcox Test at 0.01 level
Analytical	38.646	50.968	Greater	Significant (p < 2.2e-16)
Authentic	77.01	60.83	Lower	Significant (p < 2.2e-16)
Emotional Tone	31.378	42.132	Greater	Significant (p < 2.2e-16)
Positive Emotions	2.0932	2.470	Greater	Significant (*p* = 4.16e-06)
Negative Emotions	2.197	1.664	Lower	Significant (p < 2.2e-16)
Anxiety	0.597	0.426	Lower	Significant (*p* = 8.46e-13)
Anger	0.296	0.231	Lower	Significant (*p* = 5.3e-05)
Sadness	0.463	0.393	Lower	Significant (*p* = 5.73e-05)
Health	3.452	2.918	Lower	Significant (*p* = 5.56e-14)
Drives	5.363	7.22	Greater	Significant (p < 2.2e-16)
Affiliation	1.261	1.439	Greater	Not Significant (*p* = 0.58)
Power	0.68	0.56	Lower	Not significant (*p* ≈ 1.0)
Achievement	0.971	1.912	Greater	Significant (p < 2.2e-16)
Reward	1.228	1.427	Greater	Not Significant (*p* = 0.07)
Risk	0.665	0.682	Greater	Not Significant (p ≈ 1.0)
Motion	1.779	1.755	Lower	Not Significant (*p* = 0.189)
Space	5.559	5.258	Lower	Significant (*p* = 0.003)
Tentative	4.441	3.959	Lower	Significant (*p* = 6.87e-11)
Certainty	1.429	1.189	Lower	Significant (*p* = 4.42e-10)

Examining the results of the work/nonwork analysis of Epilepsy Forum posts, we find that their work-related posts are high in analytical (i.e. formal and logical) and emotional (positive and upbeat) tone than non-work posts. Examining the emotions in further detail, we find more positive emotions around work and significantly lower negative emotions and anxiety than for non-work posts. We also find more significant and positive relationships with motivation (drive), and affiliation. They are significantly lower in their focus on health. We find fewer differences around motion and space. PWE posts around work are more tentative and express less certainty, as in our first analysis with benchmark corpora.

In our analysis of a subset of small posts on discrimination, the two authors coded the posts into one of 4 categories: getting a job, workplace conditions, keeping the job and unclassified. For each post, we identified the main and primary concern in the post and assigned it to one of the four categories above. We achieved an interrater reliability of 0.67 or higher for each category. We found that PWE raised questions such as not being able to get job offers because of seizures, not getting time off for medical appointments and fear of job loss from depression. The results of our analysis of discrimination posts are presented in Table 4.

**Table 4:** Analysis of discrimination posts.

Category	Interrater reliability	Number of posts	Sample post
Getting a job	0.71	6	I was offered a job and after passing my physicals and receiving my dot card I was told I didn't qualify because I fainted over two years ago. …They had me get an eeg and see my doctor three times and spend over one thousand on it. All test and exams were normal. Is this discrimination or r they right to say i'm a safety issue. What can i do?
Workplace conditions	0.88	15	My work always gives me a hard time about taking the time for appointments (my boss)…Whenever any of my co-workers need time off or to adjust their schedule my boss is quick to accommodate them. Some of them aren't held to the same schedule and don't even work a full 8 h day as I am required to.Â They are also allowed to adjust their hours or make up time, where I have to take time out of my PTO bank. This is even if I make up the hours and end up workingÂ over 40 h a week (I'm an exempt employee). Plus my work load is higher than the others, and I get very stressed out.
Keeping the job	0.67	6	I have had epilepsy for 21 yrs. and am going thru major depression right now. I am about to lose my job due to outsourcing and I have lost my previous job due to discrimination over my epilepsy after 19 yrs. and 9 months.If there are any suggestions out there could really use some support.
Unclassified	0.97	43	

## 4 Discussion

In this study, we sought to understand the work-related concerns, motivations and emotions of PWE using unobtrusive measures to hear from PWE without fear or bias. Our cross disciplinary review of the literature on epilepsy and work indicates that others, rather than those with epilepsy, have driven perspectives on the workplace for PWE. But by studying anonymous posts on the Epilepsy Forum over a period of 16 years, we were able to extrapolate some themes regarding the employment of PWE to discuss HR initiatives that can be taken to support their workforce participation.

The conversations we overheard on the epilepsy forum are decidedly more personal, informal and less analytical. The emotional tone is more negative expressing anxiety, sadness and anger than conversations we would overhear in other areas such as Twitter. As we could anticipate, the conversations center around the bodily (neurological) issues they deal with and their health, with many posts on the relative efficacy of different medications. They disclose more anxiety as they share their problems and look for empathy, advice, and support from similar others or those familiar with epilepsy in a safe forum.

Many posts discussed how to manage risks including disclosure, sometimes when experiencing seizures in front of people, and fear of consequences. Compared to general benchmarks, PWE posts seem less ambitious or driven by their need for achievement, affiliation, and a search for rewards in their overall lives. Aligning this to previous research, we could refer to the internalization of stigma and, consequently, lower aspirations [15,27]. The tentativeness and lack of certainty in their posts create a profile of reduced confidence and self-efficacy [15].

However, as we focus on conversations that are more specific to work, we find that work has positive meaning and is associated with happiness, love and feeling good. Compared to their nonwork lives, working seems to bring a level of happiness and significantly less anxiety, sadness and anger. Work conversations tend to center more around affiliation (allies, friendships, social elements), implying that the social context of work is important to PWE. As Jacoby et al. [15] found, work provides status and independence. However, again, we find tentativeness and less certainty around work, suggesting lower self-efficacy or fragility in the workplace. Constant vigilance around seizure management and possible risks to social relationships can be stressful.

Our findings have implications for public policy and organizational practices. They highlight that psychological safety is as crucial as the physical safety delineated in government regulations and recommendations. For instance, organizational spaces and motion were of lower concern. Given that many of the accommodations listed by U.S. government agencies such as the EEOC and AskJan relate to modifications of space to reduce the occurrence of seizures and the safety of the individual if they were to occur, we had expected more concerns here. For example, driving a car is typically a concern for PWE that was present in their posts but was not picked up in the sentiment analysis.

We speculate that some of these physical accommodations are relatively easy to address in the workplace and are taken care of, so they are not as salient as we anticipated. While physical barriers to safety and success may need resources and structural modifications, many of the concerns raised by people with epilepsy are psychological and can be addressed with training to increase understanding, empathy and support. Our findings point to the continued salience of the psychological (affective) process and motivation for PWE in the workplace.

We recommend that HR managers look at the psychological components of accommodation that receive less attention than environmental changes. For instance, we have PWE posts about managers requiring them to take PTO for doctor's visits while giving other employees more flexibility for personal chores or refusing to adjust schedules that would increase the chance of seizures. Both these post examples are addressed in the EEOC accommodation guidelines but were not provided to PWE here. Providing psychological support requires an understanding of the illness and willingness to adjust on the part of managers and peers and is sometimes harder to do than changing the physical environment. We suggest that relatively low-cost investment in education on disability and training to provide managers and peers with tools to be supportive, such as having clear expectations, open communications rather than stereotyping and inclusive practices, will build a positive culture for PWE in the workplace.

In addition, we can reflect on four LWIC dimensions of anxiety, risk, body and health that are relevant to the workplace. Our sample scored significantly higher on all dimensions. Anxiety is persistent fear or worrying about everyday situations. Examining the posts scored high on anxiety, we find PWE asking about seizure fears even when they are under control, with the overwhelming dread of being outed at work; much on stress-increasing seizures; and of panic attacks from worrying about having seizures. Psychological support from managers can assuage some of these fears. Some of the accommodation tools listed by AskJan can also reduce these fears such as having a seizure plan (which requires psychological safety and disclosure). The dimension of risk relates to perceptions of danger and things to avoid. Here some of the AskJan accommodations regarding photosensitivity, balance, and fatigue are of value. Posts on body (aches, fatigue, shakes) and health can also be addressed by accommodations listed in AskJan such as a place to rest post seizure, time to take medicines and time to visit the doctor. All these will make work safer and less dangerous for PWE.

In our evaluation of a smaller subset of posts that include the word “discrimination”, we find a preponderance of posts where PWE are asking for advice on potential discrimination. These appear discriminatory since they refer to situations where PWE are treated differently from others when asking for time off for medical reasons, for adjustments of their schedule, report they don't get offers after disclosure of epilepsy, or are being fired for missing work after a seizure. Some posts describe the negative effects of organizational response to individual choices to disclose or conceal their disability. For example, the posts describe the stigma and discrimination following the disclosure of epilepsy conditions or personal distress and anxiety in obtaining jobs that require a physical exam following which their medical condition could be outed, resulting in rescinded job offers. These posts reflect the conundrum that PWD face with disclosure [28]. Sharing information on their disability may give them accommodations and reduce negative social perceptions, but the fear of stigmatization is real, especially for neurological conditions.

The strengths of this study lie in the analysis of a large dataset spanning 16 years of information that is almost impossible to collect by asking direct questions about stigmatized illness in traditional research settings. We were able to capture a rich collection of conversations from PWE without causing them distress or response bias because of our unobtrusive methodology. The anonymity of an open forum allowed for freedom to disclose and discuss topics of value to them authentically.

Methodologically, this study highlights the potential of unobtrusive research methods in researching invisible disabilities like epilepsy. We believe these methods can be used to better understand the sentiments and needs of people with other disabilities where stigma makes direct communications difficult and biased. Drawing from anonymous posts, we could hear the voices of those who would otherwise be silent. The techniques give us the ability to analyze large amounts of data while protecting the privacy of people with disabilities.

This study has several limitations. We used a dictionary-based approach for analyzing texts. While such an approach is useful for analyzing large amounts of texts, the techniques are still limited in their ability to disambiguate and understand context or relationships between constructs. However, the field is rapidly evolving, and we believe future work can build on the results in this paper by using more advanced artificial intelligence-based techniques to build a better understanding of forum posts. Our research is exploratory and innovative in that we are working with unobtrusive techniques that provide the perspective of people with epilepsy in their own voices. Building on our findings and the limitations of the study we also suggest that further research with other public data from social media like X, or digital communities like Reddit will add to a deeper understanding of PWE at work. Some of our posts were very short and the dialog in these media will allow for richer communication to analyze.

## 5 Conclusion

This study sought to uncover the work-related sentiments of people with disabilities in their own voices. Epilepsy is an invisible disability wherein first-person accounts are difficult to uncover because of stigma. Hence, the discussion on accommodation, inclusion and access for PWE has been driven by others such as managers, or caregivers and families, rather than people with epilepsy themselves. Through unobtrusive analysis of a large dataset of anonymous public discussions on the Epilepsy Forum we were able to provide new data driven insights into the workplace experiences and sentiments of PWE, to identify their areas of concern and link them to accommodations that can be made at work.

Our findings reveal the duality of work for PWE, as both a key source of identity, independence and well-being while also a source of fear from disclosure and stigma [4,15,16]. People with epilepsy expressed anxiety, health related concerns and awareness of the risks in employment and disclosure of their illness, yet their work-related discourse was more positive, affiliative and analytical. We conclude that the fear verbalized by people with epilepsy can be mitigated by increasing psychological safety and managerial support and reducing stigma with more training and awareness of epilepsy in the workplace. On a positive note, the fears around the management of their medical issues at work can be reduced with reasonable accommodations that are documented by government agencies, as well as advocacy organizations like the Epilepsy Foundation. The fears around disclosure and the social consequences of this, such as failure to get interviews or work, are more difficult to dispel. They require firms to go beyond compliance with policy to build cultures of empathy and trust where PWE can disclose without stigma and penalty.

We deliberately focused on one disability in this study because many diverse conditions fall within the disability umbrella. By grounding in one disability, we were able to have greater clarity on the unique challenges of epilepsy. For instance, we found that PWE noted both the physical support often needed in most disability accommodation, and the psychological. Their posts on unfair treatment in scheduling and performance expectations parallel those of workplace discrimination reported in prior research over the past two decades that continue [8,28]. This is important to note on two counts. One, physical changes such as ramps and chairs seem easier to make. Psychological and behavioral changes of people are more difficult. Two, PWE sentiments on whether the accommodation will be granted affect their willingness to ask for it. When firms build a supportive culture and train managers to understand disabilities, PWE are more likely to ask and receive relevant accommodations and perform better at work.

## CRediT authorship contribution statement

**Asha Rao:** Writing – original draft, Validation, Investigation, Formal analysis, Conceptualization. **Surendra Sarnikar:** Writing – review & editing, Validation, Methodology, Data curation.

## Declaration of competing interest

The authors declare that they have no known competing financial interests or personal relationships that could have appeared to influence the work reported in this paper.

## Figures and Tables

**Table:** 

**Analytical**: “Curious about epilepsy medication costs in the US compared to the UK, where government subsidies make a significant difference in treatment expenses.”
**Emotional Tone**: “Does anyone experience severe emotional symptoms like depression or intense emotional pain? It's overwhelming.”
**Affective Processes**: “I've been very depressed and need support. Can someone cheer me up with kind words or a hug?”
**Positive Emotions**: “I'm one year seizure-free today! Thank you all for your incredible support. You know who you are!”
**Negative Emotions**: “Struggling with emotional symptoms like terrible depression and overwhelming emotional pain. Anyone else?”
**Anxiety**: “Anyone taking antidepressants or anti-anxiety meds for epilepsy? I'm scared of the risks. Has anyone tried Zoloft, Lexapro, or Paxil?”
**Anger**: “Someone selfishly complained about hating life. Guess what? Most of us do. We're sick of seizures and endless medication.”
**Sadness**: “Had a seizure last night and had to call an ambulance. Feeling alone.”
**Biological**: “Does exercise affect medication levels? If someone starts exercising, will it impact their stable dilantin levels?”
**Body**: “Anyone experience heartbeat or pulsating sensations in various body parts? Has anything helped relieve it?”
**Health**: “Does anyone have another illness along with epilepsy? Were you diagnosed as a child or adult? Please share your experience.”
**Drives**: “Join me for a special Mom's Day Chat tomorrow at 4 pm East Coast time!”
**Affiliation**: “I'm a Temporal Lobe Epilepsy patient in California wanting to start a support group to share experiences and support each other.”
**Achievement**: “Has long-term seizure freedom improved emotional stability? I've found optimism and a career path, reducing my seasonal depression.”
**Reward**: “I'm fortunate to have epilepsy controlled by medication.”
**Risk**: “Planning to travel soon. Any advice or experiences, good or bad, about traveling with epilepsy?”
**Motion**: “Anyone else struggle with directions while driving? I get lost and take wrong turns easily.”
**Space**: “Anyone in their mid-late 20s in San Diego area interested in meeting to discuss epilepsy? Haven't found much yet.”
**Tentative**: “Does the Ketogenic Diet work for adults with epilepsy? Any nutritional suggestions?”
**Certainty**: “Thanks to everyone who helped me with medication issues. I'm coming off some meds with mild withdrawal but staying positive.”
**Work**: “Has anyone avoided specific careers due to epilepsy? I know I can't pursue jobs requiring long shifts, like firefighting or piloting.”

## Footnotes

1 https://www.epilepsy.com/forum-topics
